# Supplementary material for: Ecological implications of gene regulation by TfoX and TfoY among diverse Vibrio species
Source: Environ Microbiol. 2019 Mar 12;21(7):2231–47. doi: 10.1111/1462-2920.14562 (PMC6618264; doi:10.1111/1462-2920.14562)
Supplement: Supplementary file 1 — Table S1. Bacterial strains and plasmids used in this study. Fig. S1. TfoX‐ and TfoY‐induced T6SS production is conserved in pandemic V. cholerae strains. Interspecies killing assay between diverse V. cholerae strains and E. coli as prey. The V. cholerae O1 El Tor strains tested are as follows: A1552, N16961rep (with repaired frameshift mutation in hapR), C6709, E7946 and P27459 as indicated below the graph. Co‐culturing with E. coli occurred on LB agar plates supplemented with arabinose to induce tfoX or tfoY where indicated. The parental strains without inducible copies of the regulatory genes served as a control. Prey recovery is indicated as CFU/ml on the Y‐axis. Bar plots represent the average of three independent biological replicates (± SD). Statistical significance is indicated (*p < 0.05; **p < 0.01; ***p < 0.001; ****p < 0.0001). Fig. S2. TfoY production is translationally but not transcriptionally controlled by c‐di‐GMP in V. cholerae. V. cholerae reporter strains carrying a gene encoding for a translational fusion between TfoY and mCherry (tfoY‐mCherry) with or without a downstream transcriptional reporter gene (gfp) at the gene's native chromosomal locus were genetically manipulated to insert inducible copies vdcA or cdpA into their genome (inside a mini‐Tn7 transposon). Cells were then grown under inducible conditions to increase or decrease intracellular c‐di‐GMP concentrations, as shown by the arrows above the images. Detection of TfoY‐mCherry and GFP occurred through western blotting. The reporter strain lacking the transposon as well as the parental WT served as controls. Detection of σ70 served as a loading control. [file EMI-21-2231-s001.docx]

**Supporting Information**

**Ecological implications of gene regulation by TfoX and TfoY among diverse *Vibrio* species**

**Lisa C. Metzger^*^, Noémie Matthey^*^, Candice Stoudmann, Esther J. Collas,**

**Melanie Blokesch^#^**

**Supporting figures and legends**

**Figure S1: TfoX- and TfoY-induced T6SS production is conserved in pandemic *V. cholerae* strains.** Interspecies killing assay between diverse *V. cholerae* strains and *E. coli* as prey. The *V. cholerae* O1 El Tor strains tested are as follows: A1552, N16961rep (with repaired frameshift mutation in *hapR*), C6709, E7946, and P27459 as indicated below the graph. Co-culturing with *E. coli* occurred on LB agar plates supplemented with arabinose to induce *tfoX* or *tfoY* where indicated. The parental strains without inducible copies of the regulatory genes served as a control. Prey recovery is indicated as CFU/ml on the Y-axis. Bar plots represent the average of three independent biological replicates (± SD). Statistical significance is indicated (**p* < 0.05; ***p* < 0.01; ****p* < 0.001; *****p* < 0.0001).

**Figure S2: TfoY production is translationally but not transcriptionally controlled by c-di-GMP in *V. cholerae*.** *V. cholerae* reporter strains carrying a gene encoding for a translational fusion between TfoY and mCherry (*tfoY-mCherry*) with or without a downstream transcriptional reporter gene (*gfp*) at the gene’s native chromosomal locus were genetically manipulated to insert inducible copies *vdcA* or *cdpA* into their genome (inside a mini-Tn7 transposon). Cells were then grown under inducible conditions to increase or decrease intracellular c-di-GMP concentrations, as shown by the arrows above the images. Detection of TfoY-mCherry and GFP occurred through western blotting. The reporter strain lacking the transposon as well as the parental WT served as controls. Detection of σ^70^ served as a loading control.

**Table S1: Bacterial strains and plasmids used in this study.**

| **Strains or plasmids** | **Genotype*/description** | **Internal strain #** | **Reference** |
| --- | --- | --- | --- |
| **Strains** | | | |
| ***V. cholerae*** | | | |
| A1552 (WT) | Wild-type, O1 El Tor Inaba; Rif^R^ | MB_1 | ([Yildiz and Schoolnik, 1998](#_ENREF_16)) |
| A1552-lacZ-Kan | A1552 strain with aph cassette in lacZ gene; Rif^R^, Kan^R^ | MB_135 | ([Marvig and Blokesch, 2010](#_ENREF_5)) |
| A1552-Tn*tfoX-strep* | A1552 containing mini-Tn7-*araC*-P_BAD_-*tfoX-strep*; Rif^R^, Gent^R^ | MB_3420 | ([Metzger *et al.*, 2016](#_ENREF_7)) |
| A1552-Tn*tfoY-strep* | A1552 containing mini-Tn7-*araC*-P_BAD_-*tfoY-strep*; Rif^R^, Gent^R^ | MB_2978 | ([Metzger *et al.*, 2016](#_ENREF_7)) |
| A1552-Tn-Cm^R^-*tfoX_VC_-strep* | A1552 containing mini-Tn7-*araC*-P_BAD_-*tfoX_VC_-strep*; Rif^R^, Cm^R^ | MB_6753 | This study |
| A1552-Tn-Cm^R^-*tfoY_VC_-strep* | A1552 containing mini-Tn7-*araC*-P_BAD_-*tfoY_VC_-strep*; Rif^R^, Cm^R^ | MB_6754 | This study |
| A1552-Tn-Cm^R^-*tfoX_VA_-strep* | A1552 containing mini-Tn7-*araC*-P_BAD_-*tfoX_VA_-strep*; Rif^R^, Cm^R^ | MB_6755 | This study |
| A1552-Tn-Cm^R^-*tfoY_VA_-strep* | A1552 containing mini-Tn7-*araC*-P_BAD_-*tfoY_VA_-strep*; Rif^R^, Cm^R^ | MB_6756 | This study |
| A1552-Tn-Cm^R^-*tfoX_VP_-strep* | A1552 containing mini-Tn7-*araC*-P_BAD_-*tfoX_VP_-strep*; Rif^R^, Cm^R^ | MB_6757 | This study |
| A1552-Tn-Cm^R^-*tfoY_VP_-strep* | A1552 containing mini-Tn7-*araC*-P_BAD_-*tfoY_VP_-strep*; Rif^R^, Cm^R^ | MB_6758 | This study |
| A1552-Tn-Cm^R^-*tfoX_VF_-strep* | A1552 containing mini-Tn7-*araC*-P_BAD_-*tfoX_VF_-strep*; Rif^R^, Cm^R^ | MB_6759 | This study |
| A1552-Tn-Cm^R^-*tfoY_VF_-strep* | A1552 containing mini-Tn7-*araC*-P_BAD_-*tfoY_VF_-strep*; Rif^R^, Cm^R^ | MB_6760 | This study |
| A1552-tfoY-mCherry | A1552 carrying *tfoY-mCherry* translational fusion (TransFLP); Rif^R^ | MB_4262 | ([Metzger *et al.*, 2016](#_ENREF_7)) |
| A1552-tfoY-mCherry-Tn-*vdcA* | A1552-tfoY-mCherry (TransFLP) containing mini-Tn7-*araC*-P_BAD_-*vdcA*; Rif^R^, Gent^R^ | MB_4324 | ([Metzger *et al.*, 2016](#_ENREF_7)) |
| A1552-tfoY-mCherry-Tn-*cdpA* | A1552-tfoY-mCherry (TransFLP) containing mini-Tn7-*araC*-P_BAD_-*cdpA*; Rif^R^, Gent^R^ | MB_4325 | ([Metzger *et al.*, 2016](#_ENREF_7)) |
| A1552-tfoY-mCherry::GFP | A1552 carrying *tfoY-mCherry* translational fusion, transcriptionally fused to *gfp* (TransFLP); Rif^R^ | MB_5055 | This study |
| A1552-tfoY-mCherry::GFP-Tn-*vdcA* | A1552-tfoY-mCherry::GFP (TransFLP) containing mini-Tn7-*araC*-P_BAD_-*vdcA*; Rif^R^, Gent^R^ | MB_6761 | This study |
| A1552-tfoY-mCherry::GFP-Tn-*cdpA* | A1552-tfoY-mCherry:: GFP (TransFLP) containing mini-Tn7-*araC*-P_BAD_-*cdpA*; Rif^R^, Gent^R^ | MB_6762 | This study |
| N16961rep | N16961 repaired for *hapR* (*hapR*+); Str^R^ | MB_2254 | ([Kühn *et al.*, 2014](#_ENREF_4)) |
| N16961rep-Tn*tfoX-strep* | N16961rep containing mini-Tn7- *araC*-P_BAD_-*tfoX-strep*; Str^R^, Gent^R^ | MB_4142 | This study |
| N16961rep-Tn*tfoY-strep* | N16961rep containing mini-Tn7- *araC*-P_BAD_-*tfoY-strep*; Str^R^, Gent^R^ | MB_4143 | This study |
| C6709 | *V. cholerae* O1 El Tor Inaba; isolated in 1991, Peru; Str^R^ | MB_1503 | ([Wachsmuth *et al.*, 1993](#_ENREF_14)) |
| C6709-Tn*tfoX-strep* | C6709 containing mini-Tn7-*araC*-P_BAD_-*tfoX-strep*; Str^R^, Gent^R^ | MB_4156 | This study |
| C6709-Tn*tfoY-strep* | C6709 containing mini-Tn7-*araC*-P_BAD_-*tfoY-strep*; Str^R^, Gent^R^ | MB_4157 | This study |
| E7946 | *V. cholerae* strain El Tor Ogawa; isolated in 1978, Bahrain; Str^R^ | MB_2600 | ([Miller *et al.*, 1989](#_ENREF_8)) |
| E7946-Tn*tfoX-strep* | E7946 containing mini-Tn7-*araC*-P_BAD_-*tfoX-strep*; Str^R^, Gent^R^ | MB_4162 | This study |
| E7946-Tn*tfoY-strep* | E7946 containing mini-Tn7-*araC*-P_BAD_-*tfoY-strep* Str^R^, Gent^R^ | MB_4163 | This study |
| P27459 | *V. cholerae* O1 El Tor Inaba; isolated in 1976, Bangladesh; Str^R^ | MB_1504 | ([Pearson *et al.*, 1993](#_ENREF_10)) |
| P27459-Tn*tfoX-strep* | P27459 containing mini-Tn7-*araC*-P_BAD_-*tfoX-strep*; Str^R^, Gent^R^ | MB_4158 | This study |
| P27459-Tn*tfoY-strep* | P27459 containing mini-Tn7-*araC*-P_BAD_-*tfoY-strep* Str^R^, Gent^R^ | MB_4159 | This study |
| ***V. parahaemolyticus*** | | | |
| POR1 (WT) | RIMD 2210633 Δ*tdhAS* | MB_5862 | ([Salomon *et al.*, 2013](#_ENREF_11)) |
| POR1-Tn-Cm^R^-*tfoX_VP_-strep* | POR1 containing mini-Tn7-*araC*-P_BAD_-*tfoX_VP_-strep*; Cm^R^ | MB_6763 | This study |
| POR1-Tn-Cm^R^-*tfoY_VP_-strep* | POR1 containing mini-Tn7-*araC*-P_BAD_-*tfoY_VP_-strep*; Cm^R^ | MB_6764 | This study |
| POR1Δhcp1 | POR1ΔVP1393 | MB_5863 | ([Salomon *et al.*, 2013](#_ENREF_11)) |
| POR1Δhcp1-Tn-Cm^R^-*tfoX_VP_-strep* | POR1Δhcp1 containing mini-Tn7-*araC*-P_BAD_-*tfoX_VP_-strep*; Cm^R^ | MB_6765 | This study |
| POR1Δhcp1-Tn-Cm^R^-*tfoY_VP_-strep* | POR1Δhcp1containing mini-Tn7-*araC*-P_BAD_-*tfoY_VP_-strep*; Cm^R^ | MB_6766 | This study |
| POR1Δhcp2 | POR1ΔVPA1027 | MB_5864 | Gift from D. Salomon |
| POR1Δhcp2-Tn-Cm^R^-*tfoX_VP_-strep* | POR1Δhcp2 containing mini-Tn7-*araC*-P_BAD_-*tfoX_VP_-strep*; Cm^R^ | MB_6767 | This study |
| POR1Δhcp2-Tn-Cm^R^-*tfoY_VP_-strep* | POR1Δhcp2 containing mini-Tn7-*araC*-P_BAD_-*tfoY_VP_-strep*; Cm^R^ | MB_6768 | This study |
| POR1Δhcp1Δhcp2 | POR1ΔVP1393ΔVPA1027 | MB_5865 | Gift from D. Salomon |
| POR1Δhcp1Δhcp2-Tn-Cm^R^-*tfoX_VP_-strep* | POR1Δhcp1Δhcp2 containing mini-Tn7-*araC*-P_BAD_-*tfoX_VP_-strep*; Cm^R^ | MB_6769 | This study |
| POR1Δhcp1Δhcp2-Tn-Cm^R^-*tfoY_VP_-strep* | POR1Δhcp1Δhcp2 containing mini-Tn7-*araC*-P_BAD_-*tfoY_VP_-strep*; Cm^R^ | MB_6770 | This study |
| POR1ΔtfoX | POR1ΔVP1241 (deleted using  suicide plasmid pGP704-Sac-Kan-tfoX_VP_) | MB_6771 | This study |
| POR1ΔtfoY | POR1ΔVP1028 (deleted using  suicide plasmid pGP704-Sac-Kan-tfoY_VP_) | MB_6774 | This study |
| POR1ΔtfoXΔtfoY | POR1ΔtfoX ΔVP1028 (deleted using  suicide plasmid pGP704-Sac-Kan-tfoY_VP_) | MB_6777 | This study |
| POR1ΔopaR | POR1ΔVP2516 (deleted using  suicide plasmid pGP704-Sac-Kan-opaR_VP_) | MB_6780 | This study |
| POR1ΔopaR-Tn-Cm^R^-*tfoX_VP_-strep* | POR1ΔopaR containing mini-Tn7-*araC*-P_BAD_-*tfoX_VP_-strep*; Cm^R^ | MB_6781 | This study |
| POR1ΔopaR-Tn-Cm^R^-*tfoY_VP_-strep* | POR1ΔopaR containing mini-Tn7-*araC*-P_BAD_-*tfoY_VP_-strep*; Cm^R^ | MB_6782 | This study |
| POR1-tfoY*-*sfGFP*::*mCherry | POR1 carrying a *tfoY-sfgfp* translational fusion, transcriptionally fused to *mCherry* (inserted using suicide plasmid pGP704-Sac-Kan-*tfoY_VP_*-*sfGFP-mCherry*) | MB_6787 | This study |
| POR1-tfoY-sfGFP::mCherry-Tn-Cm^R^-*cdpA* | POR1-tfoY-sfGFP::mCherry containing mini-Tn7-*araC*-P_BAD_-*cdpA*; Cm^R^ | MB_6788 | This study |
| POR1-tfoY-sfGFP::mCherry-Tn-Cm^R^-*vdcA* | POR1-tfoY-sfGFP::mCherry containing mini-Tn7-*araC*-P_BAD_-*vdcA*; Cm^R^ | MB_6789 | This study |
| POR1-tfoY-sfGFP::mCherry-Tn-Cm^R^ | POR1-tfoY-sfGFP::mCherry containing mini-Tn7; Cm^R^ | MB_6790 | This study |
| ***V. alginolyticus*** | | | |
| 12G01 (WT) | Wild-type | MB_5857 | ([Salomon *et al.*, 2015](#_ENREF_12)) |
| 12G01-Tn-Cm^R^-*tfoX_VA_-strep* | 12G01 containing mini-Tn7-*araC*-P_BAD_-*tfoX_VA_-strep*; Cm^R^ | MB_6791 | This study |
| 12G01-Tn-Cm^R^-*tfoY_VA_-strep* | 12G01 containing mini-Tn7-*araC*-P_BAD_-*tfoY_VA_-strep*; Cm^R^ | MB_6792 | This study |
| 12G01Δhcp1 | 12G01ΔV12G01_01540 | MB_5858 | ([Salomon *et al.*, 2015](#_ENREF_12)) |
| 12G01Δhcp1-Tn-Cm^R^-*tfoX_VA_-strep* | 12G01Δhcp1 containing mini-Tn7-*araC*-P_BAD_-*tfoX_VA_-strep*; Cm^R^ | MB_6793 | This study |
| 12G01Δhcp1-Tn-Cm^R^-*tfoY_VA_-strep* | 12G01Δhcp1containing mini-Tn7-*araC*-P_BAD_-*tfoY_VA_-strep*; Cm^R^ | MB_6794 | This study |
| 12G01Δhcp2 | 12G01ΔV12G01_07583 | MB_5859 | ([Salomon *et al.*, 2015](#_ENREF_12)) |
| 12G01Δhcp2-Tn-Cm^R^-*tfoX_VA_-strep* | 12G01Δhcp2 containing mini-Tn7-*araC*-P_BAD_-*tfoX_VA_-strep*; Cm^R^ | MB_6795 | This study |
| 12G01Δhcp2-Tn-Cm^R^-*tfoY_VA_-strep* | 12G01Δhcp2 containing mini-Tn7-*araC*-P_BAD_-*tfoY_VA_-strep*; Cm^R^ | MB_6796 | This study |
| 12G01Δhcp1Δhcp2 | 12G01ΔV12G01_01540ΔV12G01_07583 | MB_5860 | ([Salomon *et al.*, 2015](#_ENREF_12)) |
| 12G01Δhcp1Δhcp2-Tn-Cm^R^-*tfoX_VA_-strep* | 12G01Δhcp1Δhcp2 containing mini-Tn7-*araC*-P_BAD_-*tfoX_VA_-strep*; Cm^R^ | MB_6797 | This study |
| 12G01Δhcp1Δhcp2-Tn-Cm^R^-*tfoY_VA_-strep* | 12G01Δhcp1Δhcp2 containing mini-Tn7-*araC*-P_BAD_-*tfoY_VA_-strep*; Cm^R^ | MB_6798 | This study |
| 12G01ΔtfoX | 12G01ΔtfoX (12G01ΔV12G01_19736) | MB_6994 | This study |
| 12G01ΔtfoY | 12G01ΔtfoY (12G01ΔV12G01_20658) | MB_6995 | This study |
| 12G01ΔtfoXΔtfoY | 12G01ΔtfoXΔtfoY (12G01ΔV12G01_19736 ΔV12G01_20658) | MB_6996 | This study |
| 12G01ΔluxR | 12G01ΔluxR (12G01ΔV12G01_21538) | MB_6997 | This study |
| 12G01ΔluxR-Tn-Cm^R^-*tfoX_VA_-strep* | 12G01ΔluxR containing mini-Tn7-*araC*-P_BAD_-*tfoX_VA_-strep*; Cm^R^ | MB_6998 | This study |
| 12G01ΔluxR-Tn-Cm^R^-*tfoY_VA_-strep* | 12G01ΔluxR containing mini-Tn7-*araC*-P_BAD_-*tfoY_VA_-strep*; Cm^R^ | MB_6999 | This study |
| ***V. fischeri*** | | | |
| ES114 (WT) | Wild-type | MB_5869 | ATCC-700601; via LGC standards |
| ES114-Tn-Cm^R^-*tfoX_VF_-strep* | ES114 containing mini-Tn7-*araC*-P_BAD_-*tfoX_VF_-strep*; Cm^R^ | MB_6799 | This study |
| ES114-Tn-Cm^R^-*tfoY_VF_-strep* | ES114 containing mini-Tn7-*araC*-P_BAD_-*tfoY_VF_-strep*; Cm^R^ | MB_6800 | This study |
| ***E. coli*** | | | |
| SM10λpir | thi-1 thr leu tonA lacY supE recA::RP4-2-Tc::Mu, Kmr (λpir); Kan^R^ | MB_647 | ([Simon *et al.*, 1983](#_ENREF_13)) |
| TOP10 | F- mcrA Δ(mrr-hsdRMS-mcrBC) φ80lacZΔM15 ΔlacX74 nupG recA1 araΔ139 Δ(ara-leu)7697 galE15 galK16 rpsL(Str^R^) endA1λ^-^ | MB_741 | Invitrogen |
| TOP10-TnKan | TOP10 containing mini-Tn7-*aph*(Kan^R^); Str^R^, Kan^R^, Gent^R^ | MB_4119 | ([Metzger *et al.*, 2016](#_ENREF_7)) |
| DH5α | F^-^ endA1 glnV44 thi-1 recA1 relA1 gyrA96 deoR nupG φ80lacZΔM15 Δ(lacZYA-argF) U169 hsdR17 (r_K_^-^ m_K_^+^) phoA, λ- | MB_736 | ([Yanisch-Perron *et al.*, 1985](#_ENREF_15)) |
| S17-1λpir | Tpr Smr recA thi pro hsdR2M1 RP4:2-Tc:Mu:Kmr Tn7 (λpir); Str^R^ | MB_648 | ([Simon *et al.*, 1983](#_ENREF_13)) |
| MFDpir | MG1655 RP4-2-Tc::[ΔMu1::aac(3)IV-ΔaphA-Δnic35-ΔMu2::zeo] ΔdapA::(erm-pir) ΔrecA | MB_4662 | ([Ferrieres *et al.*, 2010](#_ENREF_2)) |
| **Plasmids** | | | |
| pBAD/myc-HisA | pBR322-derived expression vector; *araBAD* promoter (P_BAD_); Amp^R^ | MB_24 | Invitrogen |
| pBAD-*tfoX-strep* | *tfoX*_VC_ in pBAD/Myc-HisA with C-terminal Strep-tagII®, arabinose inducible; Amp^R^ | MB_3616 | ([Metzger *et al.*, 2016](#_ENREF_7)) |
| pBAD-*tfoY-strep* | *tfoY*_VC_ in pBAD/Myc-HisA with C-terminal Strep-tagII®, arabinose inducible; Amp^R^ | MB_2945 | ([Metzger *et al.*, 2016](#_ENREF_7)) |
| pBAD -*tfoX*_VP_*-strep* | *tfoX*_VP_ in pBAD/Myc-HisA with C-terminal Strep-tagII®, arabinose inducible; Amp^R^ | MB_6801 | This study |
| pBAD-*tfoY*_VP_*-strep* | *tfoY*_VP_ in pBAD/Myc-HisA with C-terminal Strep-tagII®, arabinose inducible; Amp^R^ | MB_6802 | This study |
| pBAD -*tfoX*_VA_*-strep* | *tfoX*_VA_ in pBAD/Myc-HisA with C-terminal Strep-tagII®, arabinose inducible; Amp^R^ | MB_6803 | This study |
| pBAD-*tfoY*_VA_*-strep* | *tfoY*_VA_ in pBAD/Myc-HisA with C-terminal Strep-tagII®, arabinose inducible; Amp^R^ | MB_6804 | This study |
| pBAD*-tfoX_VF_-strep* | *tfoX*_VF_ in pBAD/Myc-HisA with C-terminal Strep-tagII®,, arabinose inducible; Amp^R^ | MB_6805 | This study |
| pBAD-*tfoY_VF_-strep* | *tfoY*_VF_ in pBAD/Myc-HisA with C-terminal Strep-tagII®,, arabinose inducible; Amp^R^ | MB_6806 | This study |
| pGP704-mTn7-minus SacI | pGP704 with mini-*Tn*7 harboring *aacC1* gene; Amp^R^, Gent^R^ | MB_645 | ([Nielsen *et al.*, 2006](#_ENREF_9)) |
| pGP704-mTn*tfoX-strep* | pGP704 with mini-Tn7 carrying *araC* and P_BAD_-driven *tfoX-strep*; Amp^R^, Gent^R^ | MB_3664 | ([Metzger *et al.*, 2016](#_ENREF_7)) |
| pGP704-mTn*tfoY-strep* | pGP704 with mini-Tn7 carrying *araC* and P_BAD_-driven *tfoY-strep*; Amp^R^, Gent^R^ | MB_2941 | ([Metzger *et al.*, 2016](#_ENREF_7)) |
| pGP704-mTn-*vdcA* | pGP704 with mini-Tn7 carrying *araC* and P_BAD_-driven *vdcA*; Amp^R^, Gent^R^ | MB_2943 | ([Metzger *et al.*, 2016](#_ENREF_7)) |
| pGP704-mTn-*cdpA* | pGP704 with mini-Tn7 carrying *araC* and P_BAD_-driven *cdpA*; Amp^R^, Gent^R^ | MB_2944 | ([Metzger *et al.*, 2016](#_ENREF_7)) |
| pGP704-mTn-Cm^R^ | pGP704 with mini-*Tn*7 harboring *cat* gene; Amp^R^, Cm^R^ | MB_5427 | This study |
| pGP704-mTn-Cm^R^-*tfoX_VC_-strep* | pGP704 with mini-Tn7 carrying *araC* and P_BAD_-driven *tfoX_VC_-strep*; Amp^R^, Cm^R^ | MB_6807 | This study |
| pGP704-mTn-Cm^R^-*tfoY_VC_-strep* | pGP704 with mini-Tn7 carrying *araC* and P_BAD_-driven *tfoY_VC_-strep*; Amp^R^, Cm^R^ | MB_6808 | This study |
| pGP704-mTn-Cm^R^-*tfoX_VP_-strep* | pGP704 with mini-Tn7 carrying *araC* and P_BAD_-driven *tfoX_VP_-strep*; Amp^R^, Cm^R^ | MB_6809 | This study |
| pGP704-mTn-Cm^R^-*tfoY_VP_-strep* | pGP704 with mini-Tn7 carrying *araC* and P_BAD_-driven *tfoY_VP_-strep*; Amp^R^, Cm^R^ | MB_6810 | This study |
| pGP704-mTn-Cm^R^-*tfoX_VA_-strep* | pGP704 with mini-Tn7 carrying *araC* and P_BAD_-driven *tfoX_VA_-strep*; Amp^R^, Cm^R^ | MB_6811 | This study |
| pGP704-mTn-Cm^R^-*tfoY_VA_-strep* | pGP704 with mini-Tn7 carrying *araC* and P_BAD_-driven *tfoY_VA_-strep*; Amp^R^, Cm^R^ | MB_6812 | This study |
| pGP704-mTn-Cm^R^-*tfoX_VF_-strep* | pGP704 with mini-Tn7 carrying *araC* and P_BAD_-driven *tfoX_VF_-strep*; Amp^R^, Cm^R^ | MB_6813 | This study |
| pGP704-mTn-Cm^R^-*tfoY_VF_-strep* | pGP704 with mini-Tn7 carrying *araC* and P_BAD_-driven *tfoY_VF_-strep*; Amp^R^, Cm^R^ | MB_6814 | This study |
| pUX-BF-13 | *ori*R6K, helper plasmid with Tn7 transposition function; Amp^R^ | MB_457 | ([Bao *et al.*, 1991](#_ENREF_1)) |
| pGP704-TnKan | pGP704 with mini-Tn7 carrying *aph* (Kan^R^) gene; Amp^R^, Gent^R^, Kan^R^ | MB_4117 | ([Metzger *et al.*, 2016](#_ENREF_7)) |
| pGP704-Sac28 | Suicide vector, *ori*R6K, *sacB*; Amp^R^ | MB_649 | ([Meibom *et al.*, 2004](#_ENREF_6)) |
| pGP704-Sac-Kan | Suicide vector, *ori*R6K, *sacB*; Kan^R^ | MB_6038 | This study |
| pGP704-Sac-Kan-*tfoX_VP_* | pGP704-Sac-Kan with gene fragment resulting in a 390-bp deletion within *VP1241*; Kan^R^ | MB_6815 | This study |
| pGP704-Sac-Kan-*tfoY_VP_* | pGP704-Sac-Kan with gene fragment resulting in a 393-bp deletion within *VP1028*; Kan^R^ | MB_6816 | This study |
| pGP704-Sac-Kan-*opaR_VP_* | pGP704-Sac-Kan with gene fragment resulting in a 417-bp deletion within *VP2516*; Kan^R^ | MB_6817 | This study |
| pGP704-Sac-Kan-*tfoY_VP_-sfGFP::mCherry* | pGP704-Sac-Kan with gene fragment resulting in a transcriptional fusion of *mCherry* to fusion of *VP1028-sfgfp*; Kan^R^ | MB_6819 | This study |
| pGP704-Sac-Kan-*tfoX_VA_* | pGP704-Sac-Kan with gene fragment resulting in a 402-bp deletion within *V12G01_19736*; Kan^R^ | MB_6991 | This study |
| pGP704-Sac-Kan-*tfoY_VA_* | pGP704-Sac-Kan with gene fragment resulting in a 444-bp deletion within *V12G01_20658;* Kan^R^ | MB_6992 | This study |
| pGP704-Sac-Kan-*luxR_VA_* | pGP704-Sac-Kan with gene fragment resulting in a 456-bp deletion within *V12G01_21538*; Kan^R^ | MB_6993 | This study |

*VC numbers according to ([Heidelberg *et al.*, 2000](#_ENREF_3))

**Supporting References**

Bao, Y., Lies, D.P., Fu, H., and Roberts, G.P. (1991) An improved Tn*7*-based system for the single-copy insertion of cloned genes into chromosomes of Gram-negative bacteria. *Gene* **109**: 167-168.

Ferrieres, L., Hemery, G., Nham, T., Guerout, A.M., Mazel, D., Beloin, C., and Ghigo, J.M. (2010) Silent mischief: bacteriophage Mu insertions contaminate products of *Escherichia coli* random mutagenesis performed using suicidal transposon delivery plasmids mobilized by broad-host-range RP4 conjugative machinery. *J Bacteriol* **192**: 6418-6427.

Heidelberg, J.F., Eisen, J.A., Nelson, W.C., Clayton, R.A., Gwinn, M.L., Dodson, R.J. *et al.* (2000) DNA sequence of both chromosomes of the cholera pathogen *Vibrio cholerae*. *Nature* **406**: 477-483.

Kühn, J., Finger, F., Bertuzzo, E., Borgeaud, S., Gatto, M., Rinaldo, A., and Blokesch, M. (2014) Glucose- but Not Rice-Based Oral Rehydration Therapy Enhances the Production of Virulence Determinants in the Human Pathogen *Vibrio cholerae*. *PLoS Negl Trop Dis* **8**: e3347.

Marvig, R.L., and Blokesch, M. (2010) Natural transformation of *Vibrio cholerae* as a tool-optimizing the procedure. *BMC Microbiol* **10**: 155.

Meibom, K.L., Li, X.B., Nielsen, A.T., Wu, C.Y., Roseman, S., and Schoolnik, G.K. (2004) The *Vibrio cholerae* chitin utilization program. *Proc Natl Acad Sci USA* **101**: 2524-2529.

Metzger, L.C., Stutzmann, S., Scrignari, T., Van der Henst, C., Matthey, N., and Blokesch, M. (2016) Independent Regulation of Type VI Secretion in *Vibrio cholerae* by TfoX and TfoY. *Cell Rep* **15**: 951-958.

Miller, V.L., DiRita, V.J., and Mekalanos, J.J. (1989) Identification of *toxS*, a regulatory gene whose product enhances ToxR-mediated activation of the cholera toxin promoter. *J Bacteriol* **171**: 1288-1293.

Nielsen, A.T., Dolganov, N.A., Otto, G., Miller, M.C., Wu, C.Y., and Schoolnik, G.K. (2006) RpoS controls the *Vibrio cholerae* mucosal escape response. *PLoS Pathog* **2**: e109.

Pearson, G.D., Woods, A., Chiang, S.L., and Mekalanos, J.J. (1993) CTX genetic element encodes a site-specific recombination system and an intestinal colonization factor. *Proc Natl Acad Sci USA* **90**: 3750-3754.

Salomon, D., Gonzalez, H., Updegraff, B.L., and Orth, K. (2013) *Vibrio parahaemolyticus* type VI secretion system 1 is activated in marine conditions to target bacteria, and is differentially regulated from system 2. *PLoS One* **8**: e61086.

Salomon, D., Klimko, J.A., Trudgian, D.C., Kinch, L.N., Grishin, N.V., Mirzaei, H., and Orth, K. (2015) Type VI Secretion System Toxins Horizontally Shared between Marine Bacteria. *PLoS Pathog* **11**: e1005128.

Simon, R., Priefer, U., and Pühler, A. (1983) A broad host range mobilization system for *in vivo* genetic engineering: transposon mutagenesis in Gram negative bacteria. *Nat Biotechnol* **1**: 784-791.

Wachsmuth, I.K., Evins, G.M., Fields, P.I., Olsvik, O., Popovic, T., Bopp, C.A. *et al.* (1993) The molecular epidemiology of cholera in Latin America. *J Infect Dis* **167**: 621-626.

Yanisch-Perron, C., Vieira, J., and Messing, J. (1985) Improved M13 phage cloning vectors and host strains: nucleotide sequences of the M13mp18 and pUC19 vectors. *Gene* **33**: 103-119.

Yildiz, F.H., and Schoolnik, G.K. (1998) Role of *rpoS* in stress survival and virulence of *Vibrio cholerae*. *J Bacteriol* **180**: 773-784.
